# Exploring Associations Between Early Cognitive Impairment and Echocardiographic Markers in Middle-Aged Patients with Atrial Fibrillation and Cardiometabolic Comorbidities: A Pilot Study

**DOI:** 10.3390/clinpract16050082

**Published:** 2026-04-24

**Authors:** Borislava Atanasova, Mariya Tokmakova, Angel M. Dzhambov, Rafiela Chitak, Penka Atanassova

**Affiliations:** 1 Department of Neurology, Medical University of Plovdiv, 4002 Plovdiv, Bulgaria; penka.atanasova@mu-plovdiv.bg; 2 UMHAT “Sv. Georgi” EAD, 4002 Plovdiv, Bulgaria; mariya.tokmakova@mu-plovdiv.bg (M.T.); rafiela.chitak@mu-plovdiv.bg (R.C.); 3 First Department of Internal Diseases, Cardiology Section, Medical University of Plovdiv, 4002 Plovdiv, Bulgaria; 4 Environmental Health Division, Research Institute at Medical University of Plovdiv, Medical University of Plovdiv, 4002 Plovdiv, Bulgaria; angel.dzhambov@mu-plovdiv.bg; 5 Medical Speech Language Pathology Division, Research Institute at Medical University of Plovdiv, Medical University of Plovdiv, 4002 Plovdiv, Bulgaria

**Keywords:** atrial fibrillation, mild cognitive impairment, dementia, neuropsychological assessment, echocardiography, left atrial remodeling

## Abstract

**Objectives**: Atrial fibrillation (AF), the most common sustained cardiac arrhythmia, and cardiometabolic comorbidity, have been increasingly associated with cognitive impairment and dementia. These associations, however, remain underexplored and underappreciated in middle-aged individuals with AF. This study aimed to explore the associations of early cognitive impairment with the presence of cardiometabolic comorbidities and potential associations with echocardiographic markers in middle-aged patients with and without AF. **Methods**: Between 2023–2024, fifty-six consecutive outpatients with a diagnosis of AF aged 45–65 years underwent clinical evaluation, transthoracic echocardiography, and comprehensive neuropsychological assessment using the Montreal Cognitive Assessment (MoCA) and the Consortium to Establish a Registry for Alzheimer’s Disease battery (CERAD). A control group of 58 age group-matched individuals without known cardiometabolic disease was included in comparative cognitive analyses. **Results**: Patients with AF and cardiometabolic comorbidities demonstrated early cognitive deficits, particularly in episodic memory and visuospatial functions, detectable even in individuals with normal MoCA scores, compared with the control group. However, no associations were observed between cognitive performance and conventional echocardiographic parameters in the group with AF. **Conclusions**: This study corroborated prior evidence of an association between cardiometabolic impairment and subtle cognitive impairment, but did not identify a specific contribution of echocardiography markers. More extensive and sensitive biomarkers of left atrial structure and function may be required to detect harmful associations with subtle cognitive impairment in middle-aged individuals. Further prospective studies, with a more balanced control for comorbidities, are warranted to clarify the clinical relevance of atrial structural remodeling in this context.

## 1. Introduction

Atrial fibrillation (AF) is the most common sustained cardiac arrhythmia, with an estimated global prevalence of approximately 37.5 million individuals [1]. The burden of dementia, another major public health concern, has grown to around 55 million people worldwide [2]. The prevalence of both AF and dementia is expected to increase substantially in the coming decades, largely driven by population aging [3].

Accumulating evidence indicates a strong association between AF and cognitive impairment. Several epidemiological studies have demonstrated that AF is an independent risk factor for cognitive decline, even after adjustment for traditional vascular risk factors [4]. Data from large population-based cohorts have shown that patients with AF experience poorer cognitive outcomes compared with individuals in sinus rhythm [5]. This increased risk is further amplified in the presence of comorbid conditions such as heart failure, diabetes mellitus, and chronic kidney disease. Importantly, cognitive impairment and various forms of dementia may occur in patients with AF irrespective of other established risk factors. While AF contributes to cognitive decline, modification of cardiovascular risk factors remains a cornerstone of dementia prevention [6,7].

Mild cognitive impairment is defined as a decline in one or more cognitive domains—including attention, executive function, memory, language and visuospatial abilities—relative to previous performance, without significant impairment of daily functioning [8]. This early stage of cognitive decline represents a critical window for intervention and preservation of brain health [9]. Consequently, regular cognitive monitoring in patients with AF is essential for the timely identification and management of mild cognitive impairment.

Cognitive assessment in patients with AF is typically performed in a stepwise manner [10]. Initial cognitive screening can be conducted by clinicians in routine outpatient settings using brief, validated instruments. Individuals who screen positive should undergo more detailed neuropsychological evaluation using standardized tools such as the Montreal Cognitive Assessment (MoCA) or the Mini-Mental State Examination (MMSE) [11]. Although these instruments do not establish a definitive diagnosis, they allow risk stratification and facilitate referral for specialized assessment. MoCA has demonstrated higher sensitivity for vascular cognitive impairment than MMSE, making it particularly suitable for patients with AF. Given that patterns of cognitive dysfunction vary across dementia subtypes, complementary neuropsychological instruments are often required to detect subtle early deficits [12,13].

Structural and functional remodeling of the left atrium, commonly referred to as left atrial myopathy, frequently develops in patients with AF and has been implicated in the pathogenesis of cognitive impairment [14]. Atrial fibrosis represents a key pathological substrate and results from a complex fibrogenic process involving inflammatory activation, fibroblast proliferation, and excessive extracellular matrix deposition. Central molecular mechanisms include activation of the transforming growth factor-β1 signaling pathway and the renin–angiotensin–aldosterone system [15]. Experimental studies have demonstrated that increased activity of these pathways is associated with atrial dilatation and fibrosis, which may contribute to impaired cerebral perfusion, microembolization, and chronic inflammatory responses [16]. AF has additionally been associated with an increased risk of Alzheimer’s disease, with proposed mechanisms including cerebral hypoperfusion due to irregular cardiac rhythm, atherosclerosis, and subsequent amyloid-β deposition [17].

Some studies have further suggested sex-related differences in the incidence of AF, dementia, and stroke. While men exhibit a higher incidence of AF, women with established AF appear to have a higher long-term risk of dementia [18].

The aim of the present study was to perform a neuropsychological assessment of patients with atrial fibrillation and cardiometabolic comorbidities using selected cognitive tests for the early detection of impairment across multiple cognitive domains. Additionally, the study sought to investigate the association between conventional echocardiographic parameters of left atrial structure and function and cognitive impairment in this patient population.

## 2. Materials and Methods

### 2.1. Study Design and Participants

This study was conducted between 2023 and 2024 at the University Hospital “St. George”, Plovdiv, Bulgaria. A total of 56 consecutive outpatients with AF were recruited from the Departments of Cardiology and Neurology. All participants underwent a comprehensive clinical and neurological examination, laboratory testing, electrocardiography, transthoracic echocardiography and neuropsychological assessment.

Eligible participants were aged 45–65 years. To minimize potential confounding effects on cognitive performance, enrollment in the study required the absence of severe somatic or neurological diseases. Exclusion criteria were: history of cerebrovascular accidents; traumatic brain injury with loss of consciousness exceeding one hour; epilepsy; malignant disease; substance use disorders; major psychiatric illness; decompensated chronic diseases (cardiovascular, endocrine, renal, hepatic, or pulmonary); alcohol abuse; neurodegenerative disorders (including Parkinson’s disease, Alzheimer’s disease, and vascular dementia); or inability to provide informed consent.

All patients completed a questionnaire and underwent neuroimaging, blood tests, echocardiography and neuropsychological evaluation at baseline. One year after the initial assessment, all patients underwent repeat neuropsychological testing. For the present cross-sectional analyses, we used the baseline information as echocardiography examination was only performed at the baseline.

For selected analyses, a control group of 58 individuals without cardiovascular disease, diabetes mellitus, dyslipidemia, or chronic kidney disease was included. We re-used a subsample recruited during routine preventive examinations at the Cardiology Clinic and from ambulatory care settings [19]. None of the control participants had a history of vascular risk factors or other relevant comorbidities. Controls were included only in selected analyses due to the absence of echocardiographic data.

The study protocol was approved by the Ethics Committee of the Medical University of Plovdiv (Approval No. P-1516) on 27 June 2022. Written informed consent was obtained from all participants prior to enrollment.

### 2.2. Questionnaire and Clinical Variables

At the initial visit, participants completed a structured questionnaire administered by a trained researcher. The questionnaire collected information on sociodemographic characteristics (age, sex, ethnicity, educational level and marital status), medication use, comorbidities, weight and height (from which we calculated body mass index; BMI), years of smoking (converted to pack-years of smoking), and history of COVID-19 infection.

Symptoms of anxiety and depression were assessed using the Patient Health Questionnaire-4 (PHQ-4) [20]. Responses were rated on a four-point Likert scale ranging from 0 to 3.

Clinical markers used to compare cases and controls included fasting blood glucose, total cholesterol, triglycerides, and creatinine. These were analyzed in overnight fasting blood samples analyzed at the hospital clinical laboratory using a standard clinical chemistry analyzer. Waist circumference was measured.

### 2.3. Neuropsychological Assessment

To exclude structural brain pathology, all participants underwent either computed tomography or magnetic resonance imaging of the brain at the University Hospital “St. George” or at the Translational Neurosciences Complex, Medical University of Plovdiv. Brain imaging consisted of computed tomography and/or magnetic resonance imaging depending on availability. No clinically significant structural abnormalities were identified on routine evaluation. However, the study did not include a systematic assessment of markers of cerebral small vessel disease, such as white matter hyperintensities, lacunes, or cerebral microbleeds, which may be relevant to cognitive impairment in atrial fibrillation. Imaging findings were interpreted in a clinical context and were not assessed using standardized research rating scales for small vessel disease or silent vascular lesions.

Neuropsychological testing was performed by an experienced clinical neuropsychologist. Cognitive function was assessed using the MoCA [21] and the CERAD [22] neuropsychological battery. The MoCA assesses seven cognitive domains, with a maximum total score of 30 points, including executive function, visuospatial abilities, attention, language, abstraction, delayed recall, and orientation [23,24]. Based on MoCA scores, patients with AF were categorized into two subgroups: normal cognitive performance (≥27 points) and abnormal cognitive performance (<27 points). A higher than the commonly used (≥26 points) MoCA cut-off was applied to increase sensitivity for detecting very early and subtle cognitive changes in this relatively young and clinically active population. Previous validation studies have demonstrated that higher cut-off values may be more appropriate when the primary aim is the early detection of mild cognitive impairment, particularly in populations with high educational attainment or vascular risk factors, where the standard cut-off of <26 may underestimate early cognitive decline [25]. The cut-off value was prespecified based on the study objective of maximizing sensitivity for early cognitive changes. However, given the observed differences in educational attainment between groups, the potential influence of cognitive reserve should be considered, as individuals with higher education may compensate for early neuropathological changes. No formal education-adjusted correction was applied, which should be taken into account when interpreting group differences.

Comparisons between the MoCA-defined groups were performed using CERAD subtest scores. The CERAD battery includes the Verbal Fluency Test, Boston Naming Test, Word List Recall, Word List Recognition, and Constructional Praxis. Subtest scores were summed according to standard scoring procedures to obtain a total score with established sensitivity for distinguishing mild cognitive impairment from normal cognition [26].

### 2.4. Echocardiographic Assessment

Transthoracic echocardiography was performed at baseline in accordance with contemporary recommendations of the American Society of Echocardiography and the European Association of Cardiovascular Imaging [27,28]. Standard two-dimensional, M-mode and Doppler imaging were obtained using a commercially available ultrasound system. The evaluation focused on conventional parameters of left atrial (LA) structure and left ventricular (LV) systolic and diastolic function. LA anteroposterior diameter was measured in the parasternal long-axis view. LA volume was calculated from apical four- and two-chamber views using the biplane method of disks (modified Simpson’s method) and indexed to body surface area to obtain the left atrial volume index (LAVI).

LV end-diastolic and end-systolic volumes were measured using the biplane Simpson’s method, and left ventricular ejection fraction (LVEF) was derived automatically by the system software.

Diastolic function was assessed using pulsed-wave Doppler of transmitral inflow to measure peak early (E) and late (A) velocities, E/A ratio, and deceleration time. In patients with a clinical history of persistent AF, a rhythm-control strategy was applied, and echocardiography was performed after restoration of sinus rhythm. Therefore, transmitral A-wave measurements were obtained during sinus rhythm and reflect rhythm status at the time of echocardiographic examination rather than AF subtype classification.

Tissue Doppler imaging of the septal and lateral mitral annulus was performed to obtain early diastolic velocities (e′), and the average E/e′ ratio was calculated as an estimate of LV filling pressure [28].

Advanced imaging techniques, including speckle-tracking strain analysis and left atrial deformation imaging, were not performed in the present study.

All echocardiographic examinations were acquired and interpreted by an experienced cardiologist blinded to the neuropsychological assessment results.

### 2.5. Statistical Analysis

Data were screened for missing values and summarized using descriptive statistics. Between-group differences were assessed using Welch’s *t*-test, one-way and two-way analysis of variance (ANOVA) with Tukey’s post hoc test, and Pearson’s chi-square test, as appropriate. The differences in CERAD scores between the controls and cases with cardiometabolic comorbidities and AF, each stratified by MoCA status, which defined four comparison groups, were tested using an analysis of covariance (ANCOVA), adjusting for sex, age, education, and BMI. Since for some of the CERAD subtest scores the homogeneity of variance assumption was violated according to a significant Levene’s test, the overall and between-group differences were also tested with the Kruskal–Wallis test with Dunn’s post hoc comparisons and Bonferroni correction for multiple companions. ANCOVA was also used to test the differences in echocardiographic parameters between participants with normal and abnormal MoCA scores, adjusting for potential confounding by sex, age, education, diabetes, ischemic heart disease, chronic kidney disease, and depression symptoms (PHQ-9 total score). The post hoc power for these multivariate tests was calculated using G*Power v. 3.1.9.2 (Düsseldorf, Germany: Heinrich Heine University Düsseldorf).

Statistical analyses were performed using JASP version 0.19.2.0 (JASP Team, Amsterdam, The Netherlands) and Stata MP version 19 (College Station, TX, USA: StataCorp LLC). Statistical significance was defined as a two-tailed *p*-value < 0.05.

### 2.6. Use of AI

During the preparation of this manuscript, we used ChatGPT v.5 for the purposes of improving the readability and language of the manuscript. We have reviewed and edited the output and take full responsibility for the content of this publication.

## 3. Results

### 3.1. Participant Characteristics

The study sample consisted of 56 patients with AF, including 24 women (42.9%) and 32 men (57.1%), with a mean age of 56.9 ± 6.6 years. AF was classified as either paroxysmal (N = 29, 51.79%) or persistent (N = 27, 48.21%). Twenty-six patients experienced a first episode of AF. In patients with persistent AF, rhythm restoration was attempted by cardioversion according to generally accepted clinical standards. Echocardiographic assessment was performed in sinus rhythm after rhythm-control intervention, which allowed for the evaluation of transmitral A-wave-dependent diastolic indices in these patients. Based on MoCA scores, 26 patients demonstrated normal cognitive scores, whereas 30 patients (53.6%) scored below the predefined normal threshold. Patients with abnormal MoCA scores tended to have a lower level of formal education (Table 1).

Table 2 shows a comparison of the patients with AF and cardiometabolic comorbidities and control subjects. Cases were generally older, more likely to be male, and with lower education. In terms of cardiovascular risk factors, cases had higher glucose and creatinine levels, and lower total cholesterol levels.

### 3.2. Neuropsychological Performance

**Figure 1:**
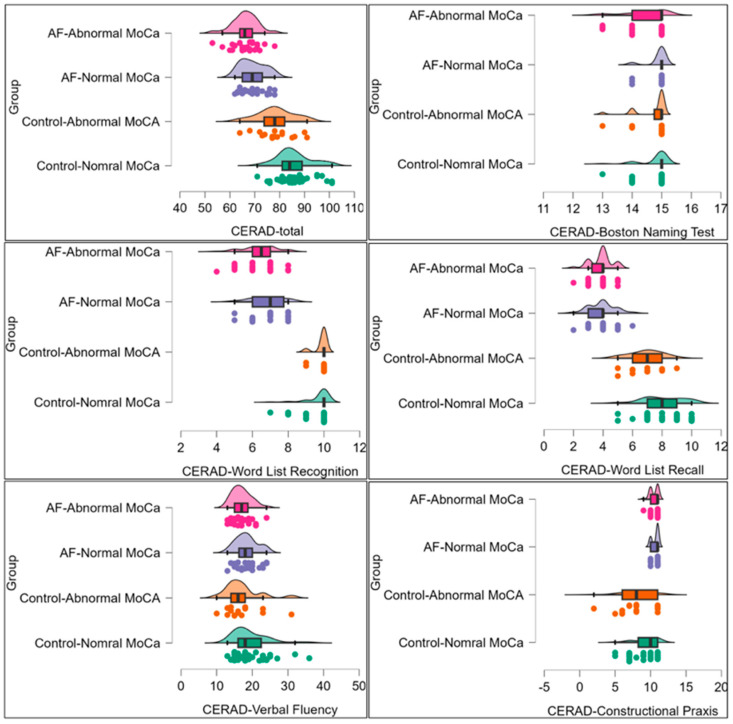
Comparison of Consortium to Establish a Registry for Alzheimer’s Disease battery (CERAD) total and subtest scores between participant groups defined by participants’ MoCA scores and AF status. Note. The differences in CERAD scores between the controls and cases with cardiometabolic comorbidities and AF were tested using an analysis of covariance (ANCOVA), adjusting for sex, age, education, and BMI. The overall and between-group differences were also tested with the Kruskal–Wallis test followed with Dunn’s post hoc comparisons and Bonferroni correction for multiple companions. Post-hoc comparisons indicate significant differences between controls and cases in CERAD-total scores, Word List Recognition, Word List Recall, and Constructional Praxis (except for the difference between the groups “controls-normal MoCA” and “AF-Abnormal MoCA”), with no differences in other sub-test scores or within the case and control groups.

Comparisons were conducted among four groups: healthy controls with normal MoCA scores, healthy controls with abnormal MoCA scores, patients with AF with normal MoCA scores, and patients with AF with abnormal MoCA scores (Figure 1). Statistically significant differences were observed in CERAD total (F _(3,104)_ = 38.54, *p* < 0.001, η^2^_p_ = 0.53) and subtest scores including the Boston Naming Test (F _(3,104)_ = 3.30, *p* = 0.023, η^2^_p_ = 0.09), Word List Recall (F _(3,104)_ = 64.05, *p* < 0.001, η^2^_p_ = 0.66), Word List Recognition (F _(3,104)_ = 78.31, *p* < 0.001, η^2^_p_ = 0.69), and Constructional Praxis (F _(3,103)_ = 12.43, *p* < 0.001, η^2^_p_ = 0.27). Conversely, there were no between-group differences in Verbal Fluency (F _(3,104)_ = 1.22, *p* = 0.307, η^2^_p_ = 0.03). In post hoc comparisons with Dunn’s test and Bonferroni correction, the controls had significantly higher CERAD-total scores, and sub-scores on Word List Recognition, Word List Recall, and Constructional Praxis (except for the difference between the groups “controls-normal MoCA” and “AF-abnormal MoCA”) than cases with cardiometabolic comorbidities and AF. The remaining CERAD subtest scores did not differ substantially between participants classified as having normal versus abnormal MoCA scores within the AF group. The post hoc power for these tests was at 80% or greater for all dependent variables, except for the Verbal Fluency scores, for which it was 31%. These findings indicate early memory and visuospatial deficits even in patients with atrial fibrillation who exhibit normal global cognitive screening results. The most pronounced impairments were observed in episodic memory, semantic memory, visuospatial abilities, and overall cognitive performance.

### 3.3. Echocardiographic Parameters Across MoCA Groups

Conventional echocardiographic parameters of left atrial structure and left ventricular function were compared between patients with normal and abnormal MoCA scores (Table 3). The number of patients included in each echocardiographic comparison was lower than the total number of patients in the MoCA subgroups for some variables because of missing or non-interpretable echocardiographic measurements. No statistically significant differences were observed in LAVI, absolute left atrial volume, left ventricular volumes, or left ventricular ejection fraction between the two groups stratified by MoCA. Exploratory analyses suggested an interaction between sex and cognitive status with respect to LVEF and LVESV, and a potential interaction between sex and cognitive status for LAVI; however, the latter finding did not reach robust statistical significance. Diastolic parameters, including transmitral E and A velocities, E/A ratio, deceleration time, and average E/e′ ratio, did not differ significantly between the groups. Overall, conventional echocardiographic measures of left atrial structure and left ventricular function were not independently associated with cognitive performance in this sample.

Adjusting the effect of MoCA group membership for potentially relevant confounding factors did not yield evidence of differences between participants with normal and abnormal MoCA scores (Table 4). However, sociodemographic characteristics and comorbidities were, in some combinations, associated with specific echocardiography parameters: older age was associated with higher average E/e′ ratio; education with higher LVEF; CKD with lower LVEF and higher LVESV; depression symptoms with lower LVEDV and lower left atrial volume; age with lower septal e′; IHD with higher left atrial volume and higher LAVI; and female sex with longer deceleration time. These models were, however, underpowered, with post hoc estimated power ranging from <1% to 41%.

## 4. Discussion

The present study found that subtle cognitive deficits in specific domains can be detected in patients with cardiometabolic comorbidity and AF even when global cognitive screening results, as assessed by the MoCA, remain within the conventionally defined normal range. Importantly, these findings should be interpreted primarily as evidence of early domain-specific cognitive impairment in middle-aged patients with AF within a cardiometabolic context, whereas the echocardiographic analyses were exploratory and did not identify robust associations. In particular, impairments in episodic memory, visuospatial abilities, and overall cognitive performance were observed in patients who scored within the normal range on the MoCA, underscoring the limited sensitivity of brief screening instruments for identifying very early cognitive changes in relatively young and clinically active populations. These findings should be interpreted within the broader context of cardiometabolic disease, as a growing body of evidence indicates that conditions such as lifestyle, arterial hypertension, ischemic heart disease, diabetes mellitus, obesity, and chronic kidney disease are independently associated with cognitive decline and an increased risk of dementia [28,29,30,31]. The underlying mechanisms are multifactorial and include chronic cerebral hypoperfusion, endothelial dysfunction, blood–brain barrier disruption, low-grade systemic inflammation, and metabolic dysregulation. Hypertension has been associated with cognitive impairment and dementia, including in middle aged patients [32]. Patients with midlife diabetes, dyslipidemia, and obesity also have an increased risk of dementia [33,34,35].

Within this cardiometabolic framework, AF may represent not only an arrhythmia but also a marker of cumulative vascular and metabolic burden contributing to early cognitive dysfunction [36]. The domain-specific impairments observed in our study suggest the need for more sensitive neuropsychological assessment strategies beyond global screening tools in patients with AF. These findings are consistent with previous studies demonstrating that patients with atrial fibrillation and cardiometabolic comorbidities may exhibit domain-specific cognitive impairment even in the absence of overt abnormalities on global cognitive screening [37,38,39]. However, the observed pattern of impairment in our study does not fully correspond to a classical subcortical vascular cognitive profile, which is typically characterized by executive dysfunction and slowed processing speed [40]. Instead, the combination of episodic memory and visuospatial deficits suggests a more heterogeneous or mixed pathophysiological substrate involving both vascular and neurodegenerative mechanisms.

Our findings could not clearly establish AF as an independent risk factor for impaired cognition due to the multiple comorbidities in the patient group. However, the evidence on this indicates that AF is a risk factor even beyond other confounding factors [41,42]. Although the present study did not demonstrate associations between cognitive performance and conventional echocardiographic parameters, accumulating evidence suggests that subclinical cardiac abnormalities may play a role in early cognitive decline [43,44]. The predominance of preserved or mildly reduced LVEF in this study further suggests that overt systolic dysfunction is unlikely to explain the observed cognitive findings. Given the limited subgroup sizes and exploratory nature of the interaction analyses, our finding of sex-specific differences should be interpreted cautiously and considered hypothesis-generating.

Structural and functional alterations of the left atrium, conceptualized as atrial cardiomyopathy, have been increasingly recognized as contributors to cerebrovascular and cognitive outcomes [45]. In our study, conventional echocardiographic indices, including E/e′ and left atrial volume, were not significantly associated with cognitive performance, suggesting that overt diastolic dysfunction is unlikely to be the primary driver of early cognitive impairment; instead, atrial remodeling may contribute through mechanisms such as cerebral hypoperfusion, microembolization, endothelial dysfunction, and chronic inflammation, supporting the concept of a “thrombogenic atrium”. Importantly, echocardiographic measurements were obtained under conditions of organized atrial contraction, which allowed for the assessment of diastolic parameters such as A-wave velocity and E/A ratio.

Large population-based cohorts, including the ARIC study, have demonstrated associations between measures of left atrial structure and function and incident ischemic stroke and dementia [46]. Importantly, more sensitive markers of atrial function, such as left atrial strain (reservoir, conduit and contractile function), LA volume, and NT-proBNP appear to show stronger associations with cognitive outcomes than structural enlargement alone [47]. Similarly, electro-physiological markers of atrial remodeling have been linked to increased dementia risk [48].

It is noteworthy that the present study used a higher MoCA cut-off (27 points) to define normal cognitive function. This may be seen as a deliberate methodological choice, but the aim was to increase sensitivity for detecting early cognitive impairment. While the original validation studies proposed a cut-off of <26 points, accumulating evidence suggests that this threshold may underestimate early or subtle cognitive deficits, particularly in middle-aged individuals and in populations at risk for vascular cognitive impairment. Thomann et al. [21] proposed a revised cut-off approach for the MoCA, demonstrating that higher thresholds improve diagnostic accuracy for early neurocognitive disorders by enhancing sensitivity, albeit at the expense of specificity. Such an approach is appropriate when the primary objective is early detection and risk stratification rather than definitive diagnosis. Support for this strategy is further provided by the findings of Gagnon et al. [25], who demonstrated that mild cognitive impairment in middle-aged adults may remain undetected by standard screening thresholds, despite clear deficits on more comprehensive neuropsychological testing. Their results highlight the vulnerability of brief cognitive screening tools to false-negative findings in populations with preserved functional independence and relatively high baseline cognitive reserve. This is particularly relevant in AF, where cognitive impairment may develop insidiously and precede overt clinical manifestations. In the present study, the appropriateness of a higher MoCA cut-off is reinforced by the observation that patients with atrial fibrillation and normal MoCA scores nonetheless exhibited significant impairments on the CERAD neuropsychological battery, particularly in episodic memory and visuospatial domains. These findings indicate that reliance on standard MoCA thresholds alone may fail to capture early cognitive decline and support the combined use of sensitive screening cut-offs and detailed neuropsychological assessment for comprehensive cognitive evaluation.

Several limitations of the present study should be acknowledged. The relatively small sample size and single-center design may have limited statistical power to detect modest associations between cognitive performance and echocardiographic parameters and may also restrict the generalizability of the findings. On the other hand, the study had sufficient power to detect differences in CERAD scores across controls and patients with cardiometabolic comorbidities and AF. In addition, the cross-sectional nature of the analysis precludes causal inference regarding the relationship between atrial fibrillation, atrial remodeling, and cognitive impairment.

Another limitation is that the control group in this study was free of known cardiometabolic disease, not just of AF, which precludes claims that AF alone was driving the lower CERAD scores in the diseased group. Therefore, the observed differences in cognitive performance should be interpreted as reflecting a combined effect of AF and cardiometabolic burden rather than AF in isolation. Moreover, the presence of comorbidities was assessed via a questionnaire, representing patient-reported medical diagnosis, which has limited sensitivity depending on the diagnosis. Furthermore, only conventional echocardiographic parameters of left atrial structure and left ventricular function were assessed, as advanced imaging techniques such as speckle-tracking-derived left atrial strain were not available. These methods may offer greater sensitivity for detecting subtle atrial dysfunction associated with cognitive decline. While transthoracic echocardiography is appropriate for routine functional evaluation, it does not fully assess all potential structural and thromboembolic mechanisms relevant to cognitive decline in atrial fibrillation. In particular, transesophageal echocardiography is the criterion standard for detecting left atrial appendage thrombi, whereas computer tomography and/or magnetic resonance imaging may provide complementary diagnostic information [49]. Therefore, conventional transthoracic echocardiography parameters alone may not fully reflect the neuroembolic substrate in this population.

AF was analyzed as a clinical entity without detailed assessment of AF burden or longitudinal rhythm variability, factors that may also affect cognitive outcomes. AF burden and ventricular rate control may influence cerebral perfusion and cognitive outcomes, but these parameters were not systematically assessed in the present study. Another potential limitation is the fact that a substantial proportion of the study population was evaluated relatively early in the course of AF. AF was classified as paroxysmal in about half of the patients, and 26 patients experienced a first documented episode. Therefore, cumulative exposure to AF-related hemodynamic disturbance, atrial dysfunction, and thromboembolic mechanisms may have been limited in part of the sample, which could have attenuated the observed associations with cognitive performance. For example, in a study of 1111 AF patients, stroke and dementia prevalence was higher in permanent AF than in paroxysmal AF [50].

Detailed information on AF duration, burden, and rhythm status at the time of neuropsychological testing was not systematically recorded and therefore could not be incorporated into the analysis. This limitation is particularly relevant, as AF burden and rhythm status may influence cerebral perfusion, thromboembolic risk, and consequently cognitive performance, potentially contributing to heterogeneity in the observed cognitive outcomes. Although efforts were made to minimize confounding, differences in educational attainment between cognitive groups may have influenced neuropsychological performance. Larger prospective studies incorporating advanced atrial functional imaging and longitudinal cognitive assessment are warranted to further clarify these relationships.

Despite these limitations, the present study suggests that comprehensive neuropsychological assessment, combined with echocardiographic evaluation, may provide insights into early cognitive impairment in patients with cardiometabolic comorbidity and atrial fibrillation, even in relatively young populations. The integration of sensitive cognitive screening with comprehensive neuropsychological testing and assessment of left atrial structural remodeling may enhance the recognition of individuals at increased risk for cognitive decline. Early detection of subtle cognitive changes could facilitate timely intervention, the optimization of cardiovascular risk factor management, and potentially improved long-term outcomes.

## 5. Conclusions

Patients with atrial fibrillation and cardiometabolic comorbidity showed lower cognitive performance across multiple domains, particularly in memory and visuospatial function. This exploratory study corroborated prior evidence of an association between cardiometabolic impairment and subtle cognitive impairment but did not identify a specific contribution of echocardiography markers. More extensive and sensitive biomarkers of left atrial structure and function may be required to detect harmful associations with subtle cognitive impairment in middle-aged individuals. Further prospective studies, with a more balanced control for comorbidities, are warranted to clarify the clinical relevance of atrial structural remodeling in this context.

## Figures and Tables

**Table 1:** Descriptive characteristics of the patients with normal and abnormal MoCA scores.

Characteristics	Normal MoCA (N = 26)	Abnormal MoCA (N = 30)	*p* ^1^
Age	56.08 ± 5.90	57.60 ± 6.20	0.351
Sex			
Male	14 (53.85%)	18 (60%)	0.643
Female	12 (46.15%)	12 (40.0%)	
Bulgarian ethnicity	26 (100%)	29 (96.67%)	0.348
Education			
Primary	0 (0%)	2 (6.67%)	0.016
High school	13 (50%)	25 (83.33%)	
Bachelor’s	6 (23.08%)	1 (3.33%)	
Master’s	6 (23.08%)	2 (6.67%)	
PhD	1 (3.85%)	0 (0%)	
Marital status			0.165
Married	23 (88.46%)	22 (73.33%)	
Divorced	0 (0%)	4 (13.3%)	
Single	2 (7.69%)	1 (3.33%)	
Widowed	1 (3.85%)	3 (10%)	
Income adequacy perception			0.431
Very easy	0 (0%)	2 (6.67%)	
Easy	4 (15.39%)	6 (20%)	
Rather easy	11 (42.31%)	7 (23.3%)	
Rather difficult	8 (30.77%)	12 (40%)	
Difficult	3 (11.54%)	3 (10%)	
Risk factors			
IHD	19 (73.1%)	21 (70%)	0.799
Hypertension	25 (96.2%)	28 (93.3%)	0.640
Diabetes	4 (15.4%)	4 (13.3%)	0.827
Dyslipidemia	7 (26.9%)	6 (20%)	0.541
CKD	1 (3.8%)	1 (3.3%)	0.918
Atrial fibrillation type			0.774
Persistent	12 (46.15%)	15 (50.00%)	
Paroxysmal	14 (53.85%)	15 (50.00%)	
CHA_2_DS_2_-VASc score	2.38 ± 0.70	2.47 ± 0.73	0.670
Anticoagulant use			0.799
No	7 (26.92%)	9 (30.00%)	
Yes	19 (73.08%)	21 (70.00%)	
Depression symptoms	3.92 ± 3.38	4.30 ± 3.41	0.681

Abbreviations: CKD—chronic kidney disease; IHD—ischemic heart disease; MoCA—Montreal cognitive assessment. ^1^ Welch’s *t*-test or Pearson’s chi-square test.

**Table 2:** Descriptive characteristics of the patients with atrial fibrillation and cardiometabolic comorbidities and controls.

Characteristics	AF-Comorbidity (N = 56)	Controls (N = 58)	*p* ^1^
Age	56.89 ± 6.06	49.95 ± 3.27	<0.001
Sex			0.015
Male	32 (57.14%)	20 (34.48%)	
Female	24 (42.86%)	38 (65.52)	
Education			<0.001
High school or lower	40 (71.43%)	17 (29.31%)	
Higher	16 (28.57%)	41 (70.69%)	
Risk factors			
Body mass index (kg/m^2^)	29.26 ± 4.80	27.84 ± 5.30	0.136
Waist circumference (cm)	92.21 ± 12.66	91.06 ± 13.10	0.633
Glucose (mmol/L)	5.76 ± 1.13	5.12 ± 0.66	<0.001
Total cholesterol (mmol/L)	5.57 ± 1.22	6.07 ± 1.12	0.027
Triglycerides (mmol/L)	1.50 ± 0.74	1.53 ± 1.36	0.901
Creatinine (µmol/L)	89.93 ± 18.08	79.95 ± 15.17	0.003
Pack years of smoking	6.79 ± 10.92	9.24 ± 11.75	0.252

^1^ Welch’s *t*-test comparisons.

**Table 3:** Comparison of conventional echocardiographic parameters across MoCA groups and sex.

Echocardiographic Parameters					Comparisons *p*-Values ^1^		
	Normal MoCA (N = 26)		Abnormal MoCA (N = 25)		Normal vs. Abnormal MoCA	Male vs. Female	MoCA × Sex
	Male (N = 14)	Female (N = 12)	Male (N = 15)	Female (N = 10)			
LAVI (mL/m^2^)	27.67 ± 7.72	25.86 ± 9.99	27.30 ± 8.09	33.50 ± 7.53	0.133	0.361	0.098
Left atrial volume (mL)	57.97 ± 17.18	50.75 ± 20.48	57.67 ± 16.71	58.11 ± 12.91	0.471	0.490	0.435
LVEF (%)	47.97 ± 7.85	55.00 ± 9.88	53.87 ± 10.50	45.83 ± 6.38	0.522	0.843	0.005
LVEDV (mL)	104.14 ± 23.50	110.75 ± 17.38	105.40 ± 24.66	111.60 ± 18.35	0.865	0.302	0.974
LVESV (mL)	54.21 ± 14.90	49.67 ± 12.74	48.20 ± 14.90	60.00 ± 9.45	0.575	0.349	0.038
E velocity (m/s)	0.75 ± 0.23	0.87 ± 0.21	0.68 ± 0.18	0.71 ± 0.16	0.059	0.190	0.514
A velocity (m/s)	0.71 ± 0.19	0.65 ± 0.32	0.64 ± 0.23	0.66 ± 0.23	0.733	0.656	0.550
E/A ratio	1.05 ± 0.54	1.43 ± 0.93	1.37 ± 0.85	1.30 ± 0.95	0.686	0.511	0.344
Deceleration time (ms)	231.57 ± 36.40	245.75 ± 110.97	205.40 ± 63.65	303.60 ± 96.32	0.483	0.016	0.067
Septal e′ (cm/s)	10.21 ± 2.15	8.98 ± 3.16	8.50 ± 3.98	9.30 ± 3.30	0.450	0.812	0.274
Lateral e′ (cm/s)	11.57 ± 2.85	10.69 ± 3.14	9.13 ± 2.92	11.20 ± 4.16	0.520	0.297	0.114
Average E/e′ ratio	7.33 ± 3.13	9.43 ± 3.80	8.42 ± 3.06	7.69 ± 3.14	0.726	0.464	0.136

Abbreviations: A velocity—late diastolic ventricular filling caused by atrial contraction; E velocity—peak speed of early mitral inflow into the left ventricle during diastole; E/A ratio—left ventricular diastolic function and filling pressure; Lateral e′—mitral annulus movement toward the apex during early diastole; LVEDV—left ventricular end-diastolic volume; LVEF—left ventricular ejection fraction; LVESV—left ventricular end-systolic volume; MoCA—Montreal cognitive assessment LAVI—left atrial volume index; Septal e′—early diastolic mitral annular velocity at the interventricular septum. ^1^ Two-way Welch’s ANOVA comparisons.

**Table 4:** Adjusted comparisons of conventional echocardiographic parameters across MoCA groups.

	Marginal Means		ANCOVA ^1^			
	Normal MoCA	Abnormal MoCA	F (df)	*p*-Values	η^2^_p_	Power
LAVI (mL/m^2^)	24.01 (17.36–30.65)	25.98 (19.23–32.74)	0.65 (1, 41)	0.425	0.02	0.17
Left atrial volume (mL)	50.56 (37.66–63.45)	52.79 (39.68–65.90)	0.22 (1, 41)	0.642	0.01	0.11
LVEF (%)	44.26 (36.98–51.55)	47.88 (40.48–55.29)	1.81 (1, 41)	0.186	0.04	0.29
LVEDV (mL)	115.56 (98.12–133.01)	119.50 (101.77–137.23)	0.37 (1, 41)	0.545	0.01	0.11
LVESV (mL)	64.55 (53.47–75.64)	62.50 (51.23–73.76)	0.25 (1, 41)	0.619	0.01	0.11
E velocity (m/s)	0.78 (0.62–0.94)	0.69 (0.52–0.85)	2.69 (1, 41)	0.109	0.06	0.41
A velocity (m/s)	0.65 (0.44–0.87)	0.65 (60.43–0.86)	0.01 (1, 41)	0.942	<0.001	<0.06
E/A ratio	1.02 (0.28–1.75)	1.13 (0.39–1.88)	0.18 (1, 41)	0.673	0.004	0.07
Deceleration time (ms)	211.47 (140.46–282.49)	223.97 (151.78–296.16)	0.23 (1, 41)	0.637	0.01	0.11
Septal e′ (cm/s)	9.39 (6.74–12.05)	9.00 (6.30–11.70)	0.16 (1, 41)	0.692	0.004	0.07
Lateral e′ (cm/s)	12.26 (9.54–14.98)	10.89 (8.12–13.66)	1.85 (1, 41)	0.181	0.04	0.29
Average E/e′ ratio	7.39 (4.68–10.09)	7.31 (4.57–10.06)	0.01 (1, 41)	0.944	<0.001	<0.06

Abbreviations: A velocity—late diastolic ventricular filling caused by atrial contraction; E velocity—peak speed of early mitral inflow into the left ventricle during diastole; E/A ratio—left ventricular diastolic function and filling pressure; Lateral e′—mitral annulus movement toward the apex during early diastole; LVEDV—left ventricular end-diastolic volume; LVEF—left ventricular ejection fraction; LVESV—left ventricular end-systolic volume; MoCA—Montreal cognitive assessment LAVI—left atrial volume index; Septal e′—early diastolic mitral annular velocity at the interventricular septum. ^1^ Analysis of covariance adjusting the effect of MoCA group membership for sex, age, education, diabetes, ischemic heart disease, chronic kidney disease, and depression symptoms.

## Data Availability

The data presented in this study are available from the corresponding author upon reasonable request.

## Abbreviations

The following abbreviations are used in this manuscript:

A velocity	Late diastolic ventricular filling caused by atrial contraction
AF	Atrial fibrillation
ANCOVA	Analysis of covariance
ANOVA	Analysis of variance
BNT	Boston Naming Test
CERAD	Consortium to Establish a Registry for Alzheimer’s Disease battery
CKD	Chronic kidney disease
E velocity	Peak speed of early mitral inflow into the left ventricle during diastole
E/A ratio	Left ventricular diastolic function and filling pressure
Lateral e′	Mitral annulus movement toward the apex during early diastole
LAVI	Left atrial volume index
LVEDV	Left ventricular end-diastolic volume
LVEF	Left ventricular ejection fraction
LVESV	Left ventricular end-systolic volume
MMSE	Mini mental state examination
MoCA	Montreal cognitive assessment
PHQ-4	Patient Health Questionnaire-4
Septal e′	Early diastolic mitral annular velocity at the interventricular septum
